# Insights into the Anaerobic Biodegradation Pathway of *n*-Alkanes in Oil Reservoirs by Detection of Signature Metabolites

**DOI:** 10.1038/srep09801

**Published:** 2015-05-13

**Authors:** Xin-Yu Bian, Serge Maurice Mbadinga, Yi-Fan Liu, Shi-Zhong Yang, Jin-Feng Liu, Ru-Qiang Ye, Ji-Dong Gu, Bo-Zhong Mu

**Affiliations:** 1 State Key Laboratory of Bioreactor Engineering and Institute of Applied Chemistry, East China University of Science and Technology, Shanghai, P.R. China; 2 Shanghai Collaborative Innovation Center for Biomanufacturing Technology, Shanghai 200237, P.R. China; 3 School of Biological Sciences, The University of Hong Kong, Pokfulam Road, Hong Kong, P.R. China

## Abstract

Anaerobic degradation of alkanes in hydrocarbon-rich environments has been documented and different degradation strategies proposed, of which the most encountered one is fumarate addition mechanism, generating alkylsuccinates as specific biomarkers. However, little is known about the mechanisms of anaerobic degradation of alkanes in oil reservoirs, due to low concentrations of signature metabolites and lack of mass spectral characteristics to allow identification. In this work, we used a multidisciplinary approach combining metabolite profiling and selective gene assays to establish the biodegradation mechanism of alkanes in oil reservoirs. A total of twelve production fluids from three different oil reservoirs were collected and treated with alkali; organic acids were extracted, derivatized with ethanol to form ethyl esters and determined using GC-MS analysis. Collectively, signature metabolite alkylsuccinates of parent compounds from C1 to C8 together with their (putative) downstream metabolites were detected from these samples. Additionally, metabolites indicative of the anaerobic degradation of mono- and poly-aromatic hydrocarbons (2-benzylsuccinate, naphthoate, 5,6,7,8-tetrahydro-naphthoate) were also observed. The detection of alkylsuccinates and genes encoding for alkylsuccinate synthase shows that anaerobic degradation of alkanes via fumarate addition occurs in oil reservoirs. This work provides strong evidence on the *in situ* anaerobic biodegradation mechanisms of hydrocarbons by fumarate addition.

Petroleum (crude oil) is a complex mixture containing thousands of chemicals, mainly of hydrocarbons, making up 80% of the oil chemical constituents. Hydrocarbons are relatively less reactive due to strong and localized C–C bond, and lack of reactive functional group^1^,^2^. Microorganisms can use hydrocarbons as the sole source of carbon and energy under either aerobic or anaerobic conditions^3^,^4^,^5^,^6^,^7^. The current understanding is that the majority of the oils degraded are a result of the activities of anaerobes living in subsurface environments under anaerobic conditions^8^,^9^,^10^. Indeed, diverse physiological groups of microorganisms (fermentative, iron-, nitrate-, sulfate-reducers, syntrophs, and methanogens, for instance) have been isolated from and/or detected in production fluids of oil reservoirs^2^,^11^. Anaerobic enrichment cultures capable of degrading and converting hydrocarbons and crude oils were also established with oil reservoir production fluids^12^,^13^,^14^,^15^,^16^,^17^. The research provides new insights into the biochemical capabilities of microorganisms originally from oil reservoirs and their involvement in the anaerobic degradation of oil hydrocarbons via detection of genes encoding for specific metabolic pathways (i.e., PCR amplification of genes *assA/masD* and *bssA* encoding for the fumarate addition pathways^12^,^13^,^14^,^15^,^16^,^18^) (for review see Refs. 19,20). Knowledge about biodegradation of petroleum hydrocarbons in oil reservoir systems will advance our understanding of the biochemical processes catalyzed by specific microorganisms and also enrich the information on the global geobiochemical carbon cycle. Sulfidogenic and denitrifying bacterial strains, such as *Desulfatibacillum alkenivorans* AK-01 and *Aromatoleum* HxN1 serve as models to investigate the fumarate addition biochemical pathway and alkylsuccinate synthase (or methylalkylsuccinate synthase) in the metabolism of oil hydrocarbons^21^,^22^,^23^,^24^. The biochemical pathways are well documented and described here in Fig. 24-30. In the fumarate addition pathway, *n*-alkanes are initially activated by addition to the double bond of fumarate at the subterminal^21^,^25^,^26^,^27^,^28^,^30^ or terminal^31^ (with propane) carbon, producing 2-(1-methylalkyl)succinates (or 2-alkylsuccinates). This process is catalyzed by alkylsuccinate synthase. Further degradation of 2-(1-methylalkyl)succinates involves carbon skeleton re-arrangement, de-carboxylation, and β-oxidations^21^. The initial products, 2-(1-methylalkyl)succinates, are commonly considered biochemical markers indicating the occurrence of the fumarate addition mechanism in the initial step^19^. Also, some cycloalkanes, *iso*-alkanes, aromatic hydrocarbons and polycyclic aromatic hydrocarbons have been reported being degraded anaerobically via addition to fumarate (such as, the detection of 2-cyclopentylsuccinate and/or 2-benzylsuccinate indicates fumarate addition pathway occurring in the anaerobic degradation of cyclopentane or toluene, respectively)^26^,^29^,^32^,^33^,^34^,^35^,^36^. Therefore, hydrocarbon-derived succinates can be used as biochemical indicators for hydrocarbon-based metabolic process, which is a powerful piece of evidences for *in situ* biodegradation mechanisms carried out biochemically through microbial activity^19^,^37^.

Metabolite profiling^32^,^36^,^38^,^39^ and *assA/masD* gene^40^,^41^,^42^ analysis of hydrocarbons-contaminated environments showed the occurrence of succinate metabolites, indicative of *in situ* biological activities. Various hydrocarbon-derived succinate analogues with side chains ranging from C3 to C11 were found in hydrocarbon-contaminated environments according to the summary available^19^. Non-saturated fatty acids with 2 mass units less than the corresponding alkylsuccinates were also detected^36^,^38^,^39^. Thus, evidence of anaerobic degradation of alkanes in such systems is well recognized. However only a limited number of metabolite profiles has been reported directly from oil reservoir production fluids. Studies on samples from Alaska North Slope oilfield showed that C1-C4 alkylsuccinates together with putative downstream metabolites were detected in production fluids^17^,^43^.

In this work, we used a multidisciplinary approach that combines metabolite profiling and functional gene (*assA/masD*) assays to investigate the specific biochemical mechanism in production fluids of twelve oil reservoirs obtained from three distinct oilfields in China. Our results, combined with the earlier works^17^,^43^ illustrate that anaerobic degradation of alkanes via the fumarate addition pathway appears to be a common initial activation strategy in different oil reservoir systems.[Fig f1]

## Results

### Physicochemical characteristics of oil reservoir production fluids

The characteristics of the investigated oil reservoir production fluids are summarized in Table 1. GC analyses of alkanes in the 12 oil samples are also provided in Supplementary Materials, (Figs. S2.1-S2.12). The amount of Na^+^, NH_4_^+^ and Cl^−^ of samples H1 to H4 ranged from 868.1 mg/L to 1148.3 mg/L, 65.5 mg/L to 1040.3 mg/L, and 306.7 mg/L to 445.9 mg/L, respectively. The SO_4_^2−^ was from non-detectable in H3 to as high as 91.0 mg/L in H1. For samples J1 to J6, the concentration of Na^+^, NH_4_^+^, Cl^−^and SO_4_^2−^ was between 3801.4 mg/L and 9577.2 mg/L, 44.0 mg/L and 1139.9 mg/L, 4500 mg/L and 21394.5 mg/L and 82.3 mg/L and 6513.5 mg/L, respectively. For samples X1 and X2, the amount of Na^+^, Cl^−^and SO_4_^2−^ was between 4196 mg/L and 5399.0 mg/L, 2000 mg/L and 5336 mg/L, and 7.7 mg/L and 124.8 mg/L, respectively.

### Signature metabolites of anaerobic degradation of hydrocarbons in oil reservoirs

Twelve production fluids collected from three different oil reservoir systems were analyzed for the presence of specific chemical metabolites showing evidence of *in situ* anaerobic biodegradation of alkanes and the biochemical mechanism involved. The mass spectra of organic extracts after derivatization with ethanol (for non-volatile organic acids) and *n*-butanol (for volatile organic acids) were also obtained and the results are given in Table 2.

**Figure 2:**
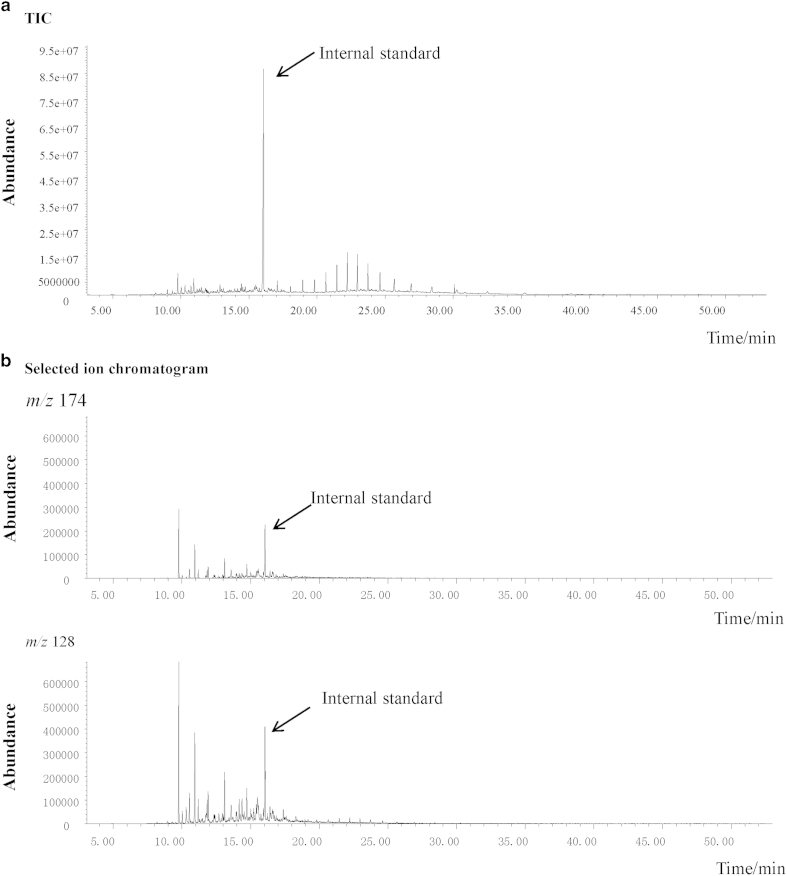
(**a**) The total ion chromatogram of fatty acid ethyl esters from production fluid H4; (**b**) Selected ion chromatogram of ethyl esters from production fluid H4 containing fragments *m/z* 174 and 128 (The most abundant peak in (**a**) is 1-chlorohexadecane that serves as the internal standard).

**Figure 3:**
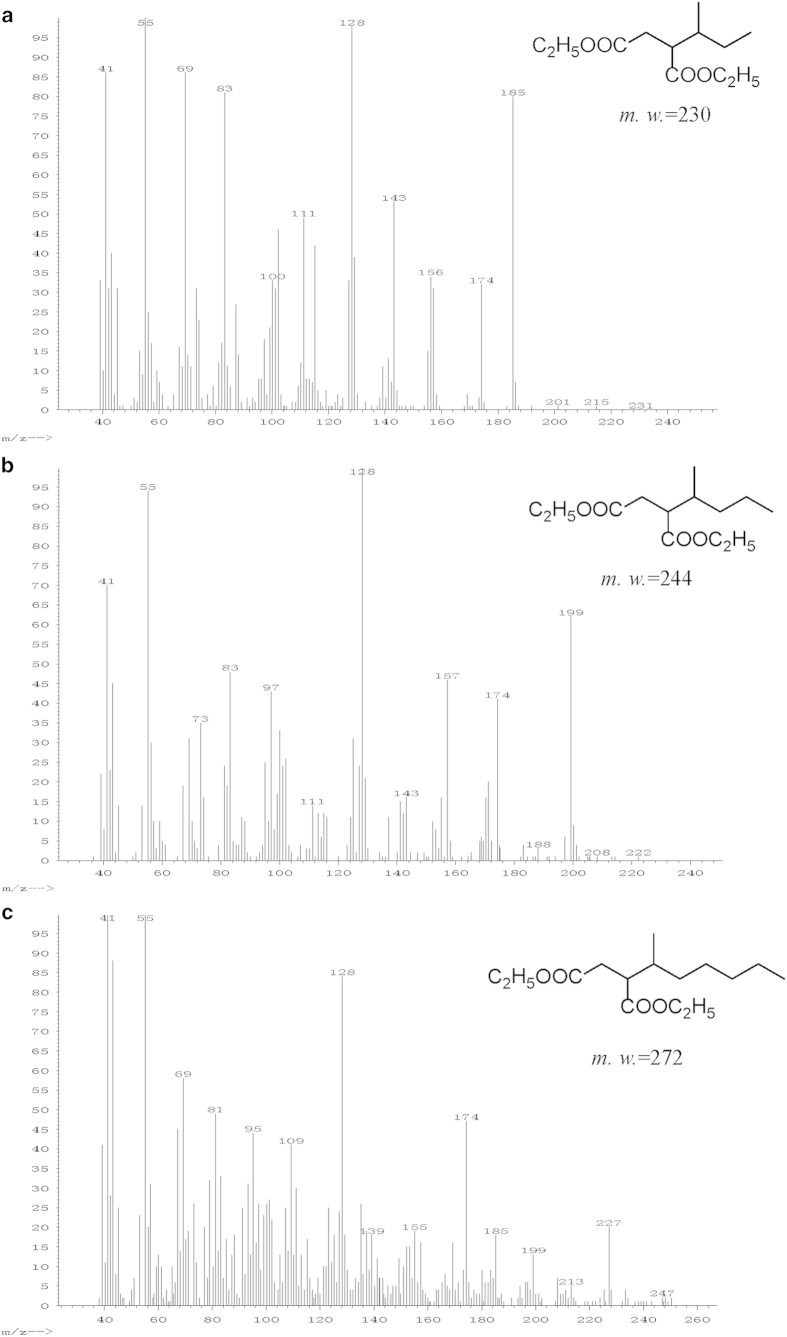
Mass spectra of some detected diethyl alkylsuccinates in sample H4.

Sample H4 was randomly chosen as an example to show its diverse alkylsuccinates and the corresponding representative mass spectra (Table 2). Fig. 2a shows the total ion chromatogram of fatty acids extracted from production fluids of H4. According to our previous study^44^, *m/z* 128 and 174 were first selected as potential indicators of alkylsuccinates and a cluster of peaks with retention time from 10.00 to 30.00 min was evident in the selected ion chromatograms (Fig. 2b). A detailed examination of the existence of fragments *m/z* M^+^-45 and M^+^-87 was also undertaken. The mass spectra of some detected diethyl alkylsuccinates in sample H4 are shown in Fig. 3. The mass spectra of other fatty acids, including diethyl and dibutyl esters, are provided in Figs. S1.13-S1. 29.

**Figure 4:**
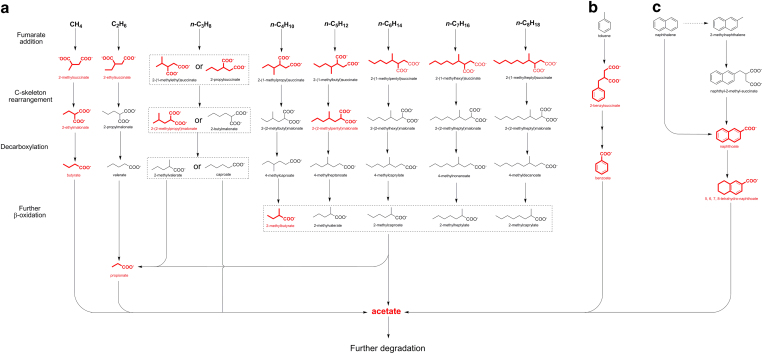
Putative anaerobic degradation pathway of hydrocarbons: (**a**) alkane, (**b**) toluene, (**c**) naphthalene or 2-methylnaphthalene. Detected organic acids are marked in red, and putative metabolites of anaerobic degradation of alkanes via fumarate addition are listed at four stages, namely fumarate addition, C-skeleton re-arrangement, decarboxylation and further β-oxidation.

Comparison of retention times and mass spectral characteristics of chemically synthesized alkyl-succinates^44^, a suite of (putative) alkyl-substituted succinates of parent *n*-alkanes with chain length from C1 to C8 (Fig. 4 and Table 2) were detected by GC-MS analysis. In addition, 2-benzylsuccinate and 2-naphthoate known as indicators of anaerobic degradation of mono- or poly-aromatic hydrocarbons were also detected in some of the samples in this study (Fig. 4b, c and Table 2).

### Putative downstream metabolites of the fumarate addition pathway

Alkylmalonates (C2, C4 and C6), known as downstream degradation intermediates of alkyl-succinates via C-skeleton rearrangement, were also observed in samples H3, H4, J2 and J4. Volatile fatty acids such as formate, acetate, propionate, butyrate, their branched derivatives and long-chain fatty acids (stearate, palmitate and myristate) were also found in some samples. These fatty acids are also produced during the anaerobic degradation of alkanes. Remarkably, 5, 6, 7, 8-tetrahydro-naphthoate as the downstream metabolite of 2-naphthoate was detected in sample H3 and J4 (Table 1). The metabolic pathways that could produce 5, 6, 7, 8-tetrahydro-naphthoate and 2-naphthoate from naphthalene and/or 2-methyl naphthalene are given in Fig. 4c^9^,^45^.

### Investigation of alkylsuccinate/2-(1-methylalkyl)succinate synthase alpha-subunit (*assA/masD*) gene

**Figure 5:**
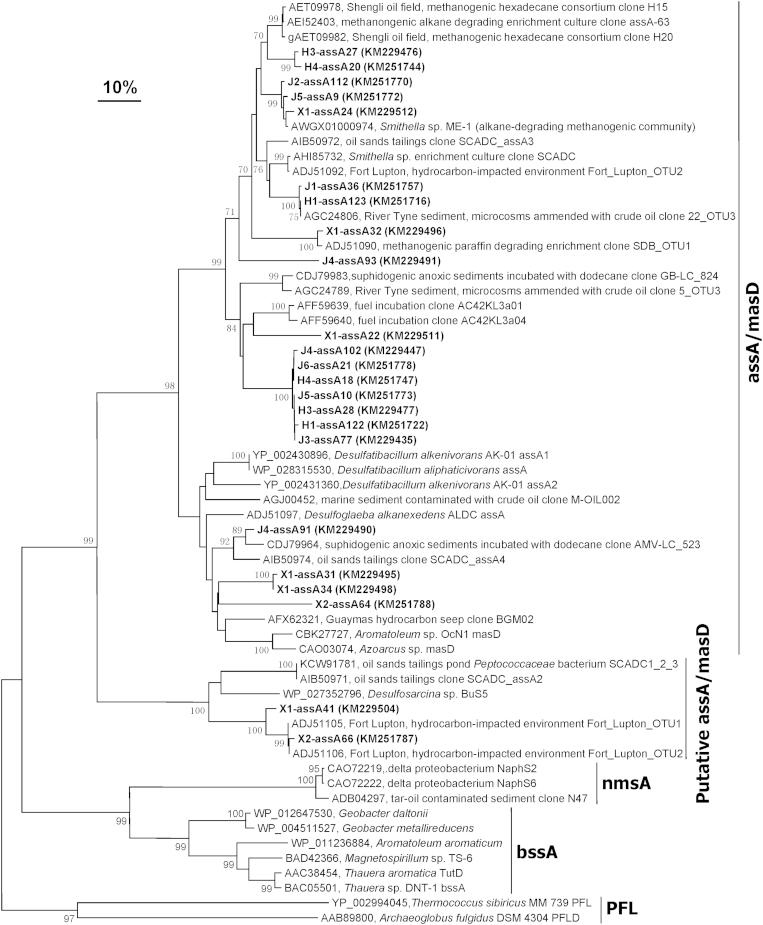
Phylogenetic tree of deduced *assA/masD* gene sequences amplified from DNAs extracted from the oil reservoir production fluid samples and closely related sequences from the GenBank database. Phylogenetic analyses were conducted with MEGA6 software. The topology of the tree shown was obtained with the neighbor-joining method and the Poisson correction. Values below 70% are not shown. The database was accessed on between July and August 2014. *Scale bar* = 10% amino acid substitution.

The detection of alkylsuccinates in most of the samples indicates that anaerobic degradation of alkanes occurs in oil reservoir systems via the fumarate addition mechanism. To provide additional evidence on the microbial communities capable of anaerobic degradation of alkanes via fumarate addition in these samples, genomic DNAs extracted from the production fluid samples were PCR amplified for the presence of functional genes (*assA*/*masD*) encoding for enzymes that initiate anaerobic degradation of alkanes. Clone libraries were then established from eleven of the twelve production fluid samples and all the cloned sequences were affiliated with *assA*/*masD* genes (Fig. 5), but no expected amplification band could be obtained from DNA extract of sample H2. Subsequent analysis was carried out at the protein level of deduced *assA*/*masD* gene sequences. OTUs H3-assA27 and H4-assA20 were 91% similar to an *assA*/*masD* gene sequence (AEI52403) obtained from a methanongenic alkane-degrading enrichment culture. OTUs H3-assA27, H4-assA20 also shared at least 85% identity with *assA*/*masD* sequence from *Smithella* ME-1^46^,^47^. OTUs J2-assA112, J5-assA9 and X1-assA24 were highly related (96-98% identity) to *assA*/*masD* sequence from *Smithella* ME-1^46^,^47^. OTUs J1-assA36 and H1-assA123 showed close relationship to *assA*/*masD* gene sequence (AGC24806) from River Tyne sediment microcosms amended with crude oil^18^. OTU X1-assA32 was a close relative (98% identity) to *assA*/*masD* gene sequence (ADJ51090) retrieved from a methanogenic paraffin degrading enrichment^40^. X1-assA22 clustered with 75% identity to *assA*/*masD* gene sequences obtained from fuel incubation^48^. J4-assA93 appeared to be far related to any *assA*/*masD* gene sequence available in the GenBank database. OTUs J3-assA77, J4-assA102, J5-assA10, J6-assA21, H1-assA122, H3-assA28 and H4-assA18 clustered together and were moderately related to the known *assA*/*masD* gene sequences; which could indicate the existence of a potentially “new clade” of *assA*/*masD* based on gene sequences (Fig. 5).

OTUs J4-assA91, X1-assA31, X1-assA34 and X2-assA64 shared at least 72 to 94% identity to *assA*/*masD* gene sequences previously detected in samples obtained from suphidogenic anoxic sediments incubated with dodecane^49^, oil sands tailings (accession number AIB50974) and sediment from a hydrocarbon seep in the Guaymas Basin, Gulf of California^41^. Finally, OTUs X1-assA41 and X2-assA66 were most closely related (90-96% identity) to *assA*/*masD* gene sequences from hydrocarbon-impacted aquifers near Fort Lupton, Colorado^40^.

## Discussion

Oil reservoirs represent specific environments in which microorganisms, especially anaerobes, have been implicated in the formation of the heavy oil that is found in geographically distinct reservoirs around the world^8^. The presence of anaerobes in such systems has led to the assumption that these microorganisms may be used to recover more energy as natural gas via methanogenic conversion of oil hydrocarbons^50^,^51^,^52^. Alkanes represent an important fraction of crude oil and in recent years, studies have been undertaken to understand the activation of these relatively less reactive compounds under anaerobic conditions and the addition of alkanes onto the double bond of fumarate with subsequent formation of alkylsuccinates as a prominent biochemical activation mechanism (for review see Ref 5). The detection of alkylsuccinates in engineered settings and/or environmental samples is indicative of the activity of microorganisms using the fumarate addition mechanism^19^,^37^. So far, alkylsuccinates have been found in anaerobic enrichment cultures amended with either alkanes or crude oil^15^,^18^ and also in environmental samples obtained from oil-contaminated sites (for review see Ref 19,37), but scarcely reported in samples originating from oil reservoirs. In the present study, metabolite profiles of samples collected from three different oil fields were analyzed using GC-MS, and at the same time, alkylsuccinates as well as putative downstream metabolite alkylmalonates were found in eleven of the twelve samples investigated in the three oilfields. Collectively, these identified metabolites are supportive for the anaerobic activation of alkanes in oil reservoirs via the fumarate addition biochemical pathway. The detection of other alkanoic acids suggests a further degradation of alkylsuccinates in the investigated environments; though these alkanoic acids can have multiple sources.

In addition, 2-benzylsuccinate and naphthoate together with 5,6,7,8-tetrahydro-naphthoate (metabolites produced during the anaerobic degradation of toluene, naphthalene and/or methyl naphthalene) were also identified in samples H3, J1, J4, J6, X1 and X2^9^,^34^,^53^. This set of metabolites identified indicates that, besides alkanes, mono- and polycyclic aromatic hydrocarbons were also degraded anaerobically in the oil reservoirs. Therefore, the putative biodegradation pathways of these organic acids were illustrated in Fig. 4a. Generally, at least one compound in each biochemical step was detected in the samples analyzed. It is noteworthy that both 2-(1-methylheptyl)succinate and 2-(methylpentyl)malonate are so far the two largest signature metabolites in terms of molecular weight from alkanes degradation observed in oil reservoirs.

Furthermore, to obtain additional evidence on the microbial communities capable of anaerobic degradation of alkanes via fumarate addition, functional genes *assA*/*masD* were PCR amplified from the oil reservoirs samples. Expected DNA bands were obtained successfully in eleven of the twelve samples, and further cloned and sequenced. The results indicate the presence in the oil reservoirs of microorganisms harboring *assA*/*masD* gene encoding for enzyme(s) that initiates anaerobic alkane degradation via fumarate addition mechanism. Combined with the detection of alkylsuccinates as signature metabolites, our data shows that anaerobic degradation of alkanes via the fumarate addition pathway occurred in the oil reservoirs. The combination of the two methods (metabolite profiling and functional gene amplification) should prove a useful and more comprehensive approach to gain insights into the anaerobic degradation of alkanes (via fumarate addition) in oil reservoirs despite of the fact the occurrence of other degradation strategies cannot be excluded^3^,^18^. For example, 2-(1-methylethyl)succinate was indeed detected in H2, but no *assA* gene products could be amplified from the DNA extracted from this sample, probably due to the specificity and coverage of PCR primers (primers sets used are not “all-inclusive” of “universal” templates) used for DNA amplification.

It is difficult, with limited information, to link the formation of alkylsuccinates in the samples analyzed with specific metabolic processes such as nitrate-, iron-, sulfate-reduction, or methanogenesis (i.e., when and how these specific biomarkers were formed). For instance, several samples (H1, H3, H4, J1, J2, J5 and X1) contained *assA/masD* gene homologues highly related to those from members of the genus *Smithella*; a genus that was implicated in the anaerobic degradation of crude oil alkanes under methanogenic conditions^46^,^47^,^54^. However, except for sample X1 (21 °C) in which alkylsuccinates were not detectable, all other samples had temperature above 37 °C; temperature beyond the optimum reported for the growth of *Smithella propionica*, the only known isolate of the genus *Smithella*^55^. Therefore, we speculate that microorganisms carrying *assA* gene similar to those from members of the *Smithella* may exist in the oil reservoirs production fluids. On the other hand, assuming that these *assA* gene sequences were truly from members of the genus *Smithella* and since, to date, relatives to this genus have not been reported to thrive in high temperature reservoirs, we speculate that they could survive in the cooler part of the reservoirs.

Oil reservoirs are large and complex environments, different from laboratory cultures in tubes and/or bottles, therefore various metabolic processes may occur in different zones (or depth) of the same reservoir. Previous work showed that anaerobic degradation of alkane (methane to butane) also occurred in the pipeline of Alaskan North Slope oil field^43^. That is, when sampling from production wells, the information from the geological formation and pipeline infrastructure may be mixed and that should also be considered when interpreting phylogenetic data.

Based on current data from the production fluids, it is impossible to obtain direct evidence on the connection between fumarate addition and methanogenic/sulfate-reducing or other relevant biochemical/physiological processes. However, using a combination of approaches involving biochemical and functional gene profiling simultaneously, this study does suggest that anaerobic degradation of alkanes via fumarate addition pathway occurs in oil reservoirs.

## Conclusion

The detection of signature biomarkers of anaerobic degradation of *n*-alkanes in conjunction with the positive detection of associated alkylsuccinate synthase genes in samples from oil reservoirs supports the occurrence of the fumarate addition pathway in oil reservoirs. Our results, in conjunction with other data, support the hypothesis that fumarate addition mechanism plays an important role in anaerobic alkane transformation in oil reservoirs.

## Materials and Methods

All chemical reagents including *n*-hexane, ethanol, cyclohexane, ethyl acetate, *n*-butanol, dodecane, chlorohexadecane, NaOH, NaHSO_4_, Na_2_SO_4_, and H_2_SO_4_ were of analytical grade and purchased from Shanghai Lingfeng Chemical Reagent Co. Ltd. (Shanghai, China).

### Site descriptions and sample collection

Production fluid samples were collected from crude-oil-producing wells in Jiangsu, Xinjiang and Huabei Oilfields, of China. The temperature was 80~90 °C (Jiangsu Oilfield, sample J1 to J6), 37~45 °C (Huabei Oilfield, sample H1 to H4) and 21 and 32 °C (Xinjiang Oilfield, sample X1 and X2). The three oil fields have coverage of low-, mesophilic-, and high-temperature reservoirs. Other physicochemical information of each oilfield is given in Table 1. The alkane composition of the samples Hs, Js, and Xs are also provided in Figs. S2.1-S2.12. The samples were collected into sterile 5 L bottles to completely full and then capped after flushing the lines for about 30 min.

### Extraction of organic acids

#### Extraction of long-chain fatty acids

Production fluid (approximately 0.5 L) from each sample was transferred into a 1 L round-bottom flask containing 2.5 g of NaOH. The contents were thoroughly mixed with a mechanical stirrer for 2 h. Then, aqueous phase was collected and extracted three times with 30 mL of *n*-hexane. The oil phase was mixed with 100 mL of 1% NaOH in 50% ethanol-water solution for 2 h, and the process was repeated once. The aqueous phase was further treated as described above and the extracts were combined. The solution was filtered, concentrated and acidified with HCl to pH < 2 at 0 °C. Ten mL of ethyl acetate were added and, organic acids extracted three times. The solvent was removed by rotary evaporation at 45 °C and the residues were dried by passing through Na_2_SO_4_.

#### Extraction of volatile fatty acids

To extract volatile fatty acids, ammonia was added to 50 mL of production fluid in a tube until pH > 10. The tube was heated at 105 °C in an oven until completely removal of water. The residues were sealed tightly before further use.

#### Derivatization of long chain fatty acids

The extracted long-chain fatty acids were derivatized via ethyl esterification. In a 100 mL round-bottom flask, a solution of ethanol-cyclohexane (10 mL, 1: 1), and 0.2 g of NaHSO_4_ was added into the organic acids extracts. The flask equipped with a water separator was then transferred to an oil-bath, and refluxed at 80 °C till no more water was produced. After cooling to room temperature, ethanol and cyclohexane were removed, and deionized water was added. Esters were extracted three times with 10 mL of ethyl acetate, and then combined. The ethyl acetate was removed after drying over anhydrous Na_2_SO_4_.

#### Derivatization of volatile fatty acids

Volatile fatty acids were analyzed after derivatization *via n*-butyl esterification as previously described in Ref. 56.

#### Gas chromatography-Mass spectrometry (GC-MS)

All GC-MS analyses were performed on an Agilent 6890 GC equipped with an HP-5MS capillary column (30 m × 0.25 mm × 0.25 μm) and a mass detector (MSD 5975). For analyses of long-chain fatty acids, the injection port temperature was held at 280 °C. The oven temperature was initially held at 60 °C for 2 min, then increased at a rate of 10 °C per min to 260 °C and held at this final temperature for 30 min. The MS detector acquired the data in the scan mode, from 30 to 1000 mass units. For volatile fatty acids, the injection port temperature was 250 °C. The oven temperature was initially held at 60 °C for 1 min, and then increased at 15 °C per min to 145 °C. The MS detector acquired data in the scan mode, from 30 to 210 mass units. EI was operated at 70 eV and the ion source temperature was held at 230 °C for both long-chain and volatile fatty acids. Analytical reproducibility for replicate analyses (*n* = 3) of the alkylsuccinate of parent alkane C1-C8 in the production fluids was 0.12% relative standard deviation.

#### Identification of alkylsuccinates/2-(1-methylalkyl)succinates

GC-MS was used for the characterization and identification of possible degradation intermediates. GC-MS is well suited for coping with high sample numbers in reasonable measurement times with respect to both technical accuracy and identification and quantification of low-molecular-weight metabolites^57^.

The identification of diethyl alkylsuccinate was performed as established previously by Bian *et al*^44^. The study revealed that diethyl alkylsuccinates have four EI mass spectrum characteristics at *m/z* 128, 174, M^ + ^-45 and M^+^-87 (Mass spectra of identified alkylsuccinates are shown in Supplementary Materials as Figs. S1.1-S1.8). Diethyl alkylsuccinates were identified by scanning the above four characteristic ions in the total ion chromatogram and comparison of the retention times with those of standard compounds^44^.

### DNA extraction

Approximately 600 ml of each production fluid were filtered onto membrane filters (0.2-μm-pore-size, 50 mm diameter, Shanghai, China). Genomic DNAs were extracted from the filters using an E.Z.N.A.™ Soil DNA kit (D5625-01, Omega Bio-Tek, Inc., USA), according to the manufacturer’s protocol.

### Amplification of alkylsuccinate/2-(1-methylalkyl)succinate synthase alpha-subunit (*assA/masD*) gene fragments

Portions of gene encoding the alpha subunit of the alkylsuccinate synthase were amplified with three primers sets, *viz*. assA2F (5′-YATGWACTGGCACGGMCA-3′)/ assA2R(5′- GCRTTTTCMACCCAKGTA-3′)^18^, 7757f-1 (5′-TCGGACGCGTGCAACGATCTGA-3′)/ 8543R (5′-TCGTCRTTGCCCCAYTTNGG-3′), and 7766f (5′-TGTAACGGCATGACCATTGCGCT-3′)/ 8543R (5′-TCGTCRTTGCCCCAYTTNGG-3′)^41^ were used for the amplification in this study. The thermal cycler program for primers assA2F/assA2R was performed as described by Aitken *et al*^18^, and primer sets 7757f-1/8543R and 7766f/8543R followed the condition described by von Netzer *et al*^41^. Unless otherwise mentioned, all PCR products obtained above were first visualized by agarose gel (1%, w/v) electrophoresis followed by gel staining (DuRed nucleic acid gel stain, Beijing, China) to ensure the correct size fragment was amplified. Subsequently, PCR products resulting from independent five (5) reactions were pooled and visualized by agarose gel (1.8%, w/v) electrophoresis (50 min at 160 V). The appropriately sized fragments were excised and purified with a DNA purification kit (Axygen® Biosciences, Inc., CA, USA) prior to cloning.

### Construction of *assA/masD* genes clone libraries, sequencing and phylogenetic analyses

Purified *assA*/*masD* gene-PCR fragments were directly cloned into *Escherichia coli* DH5α using a pMD19^®^-T Simple cloning vector (Takara®, Japan) following the instructions of the manufacturer. Recombinant cells were spread onto LB agar plates containing ampicillin, IPTG and X-Gal. White clones were randomly selected and cultured overnight at 37 °C in 0.8 ml of Luria Broth (LB) medium in the presence of ampicillin. The clones were screened for the presence of correct insert by PCR using the forward M13F (-47) (5′-CGCCAGGGTTTTCCCAGTCACGAC-3′) and the reverse RV-M (5′-GAGCGGATAACAATTTCACACAGG-3′) plasmid specific primers, followed by agarose gel electrophoresis with subsequent DuRed staining. Sequencing was performed on an ABI 3730 sequencer (Dye-Terminator Cycle Sequencing; Applied Biosystems). The obtained *assA*/*masD* gene sequences were first trimmed to remove vector sequences and then compared to GenBank Database using the BLASTX algorithm to identify nearest related ones. *assA*/*masD* gene sequences were clustered into OTUs and representative OTUs from clones libraries as well as reference sequences from GenBank were translated and aligned using Clustal Omega^58^. Phylogenetic tree was constructed based on the Neighbor-Joining method^59^ and the Poisson correction method using the MEGA6 software^60^. The percentage of replicate trees in which the associated taxa clustered together in the bootstrap test (1000 replicates) is shown next to the branches. *assA*/*masD* gene fragments obtained in this study were deposited in the GenBank database under accession numbers KM229422-KM229513 and KM251716-KM251802.

## Author Contributions

This study was designed by X.-Y.B., S.M.M., S.-Z.Y. and B.-Z.M. X.-Y.B. performed the analyses of metabolites in production fluid samples. X.-Y.B., S.M.M. and Y.-F.L prepared genomic DNAs, amplification and cloning exercises for assA/masD gene assessment. Phylogenetic analyses were performed by S.M.M. assisted by X.-Y.B. and Y.-F.L. X.-Y.B. and S.M.M. wrote the manuscript, assisted by all co-authors. J.-F.L. provided oil reservoirs data. R.-Q.Y. and J.-D.G. provided valuable suggestions in the design of the experiments and the preparation of the manuscript. All authors reviewed the final manuscript.

## Additional Information

**How to cite this article**: Bian, X.-Y. *et al*. Insights into the Anaerobic Biodegradation Pathway of *n*-Alkanes in Oil Reservoirs by Detection of Signature Metabolites. *Sci. Rep.* **5**, 9801; doi: 10.1038/srep09801 (2015).

## Figures and Tables

**Figure 1 f1:**
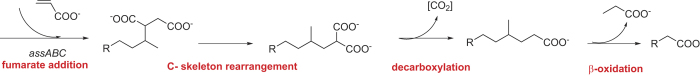
Proposed fumarate addition mechanism in anaerobic degradation of alkanes.

**Table 1:** Physicochemical parameters of the samples collected from the twelve oil reservoir samples.

	**H1**	**H2**	**H3**	**H4**	**J1**	**J2**	**J3**	**J4**	**J5**	**J6**	**X1**	**X2**
**Temperature (°C)**	**37~45**				**80~90**						**21**	**32**
Na ^+^ (mg/L)	1148.3	1173.9	868.1	962.2	9577.2	4235.3	8750.2	3909.2	3801.4	6218.7	4196	5399.0
NH_4_ ^+^ (mg/L)	375.0	65.5	1109.7	1040.3	963.9	44.0	n. d.*	1139.9	85.5	61.1	n. d.	n. d.
Cl^–^ (mg/L)	306.7	428.3	445.9	423.7	21394.5	4500	13178.7	8418.5	6825.8	12575.17	2000	5336
SO_4_^2–^ (mg/L)	91.0	80.7	n. d.	42.6	2676.2	6513.5	82.3	335.3	2050.6	2392.4	124.8	7.7

^*^n. d.: not detectable

**Table 2:** Signature metabolites of anaerobic alkanes degradation detected in production fluid samples.

	**H1**	**H2**	**H3**	**H4**	**J1**	**J2**	**J3**	**J4**	**J5**	**J6**	**X1**	**X2**
**Alkylsuccinates**												
C1								+				
C2	+		+		+		+	+	+	+		
C3	+	+	+	+	+				+	+		
C4	+		+	+					+	+		+
C5	+		+	+					+	+		+
C6	+			+								+
C7	+			+								
C8	+			+								
Benzylsuccinate										+		
**Products of the carbon skeleton rearrangement**												
Ethylmalonate				+								
Butylmalonate						+		+				
2-(methylpentyl)malonate			+									
**Metabolite of naphthalene and/or methylnaphthalene**												
Naphthoate			+		+			+			+	+
5,6,7,8-tetrahydronaphthoate			+					+				
**Alkanoate**												
Formate						+						
Acetate		+		+		+		+		+		
Propionate			+	+								
2-methylpropionate			+									
Butyrate			+			+		+		+		
1-methylbutyrate	+	+	+									
Hydroxycaproate	+							+				
Octanoate								+				
4-octenoate				+					+	+		+
3-nonenoate	+			+				+		+		+
Nonanoate										+		
Laurate								+		+	+	
9-hexadecenoate	+								+			
Myristate	+	+					+	+	+	+	+	
3-hydroxytridecanoate	+											
Palmitate	+	+	+	+	+	+	+	+	+	+	+	+
Oleate	+	+							+			
Stearate			+				+	+				+

“+”: detected.
